# An Analysis From a Tertiary Pediatric Hospital: Does Physical Activity Play a Role in the Management of Children and Young Adults With Osteogenesis Imperfecta?

**DOI:** 10.7759/cureus.53646

**Published:** 2024-02-05

**Authors:** Francisca Galhardo Saraiva, Joana Jonet, Margarida Roquette, Joana Ovídio, Mafalda S Pires, João Lameiras Campagnolo

**Affiliations:** 1 Pediatrics, Hospital Fernando Fonseca, Amadora, PRT; 2 Pediatrics, Hospital de Cascais Dr. José de Almeida, Alcabideche, PRT; 3 Pediatric Orthopedics, Hospital Dona Estefânia, Lisbon, PRT; 4 Pediatric Orthopedics, Centro Hospitalar Universitário de Lisboa Central, Lisbon, PRT; 5 Rehabilitation Medicine, Centro Hospitalar Universitário de Lisboa Central, Lisbon, PRT

**Keywords:** connective tissue disorder, physical medicine and rehabilitation (pm&r), orthopedics, pediatrics, bone fragility, pediatric fractures, pediatrics rehabilitation, exercise, physical activity, osteogenesis imperfect

## Abstract

Introduction: Osteogenesis imperfecta (OI) is a hereditary connective tissue disorder characterized by reduced bone density and increased proneness to fractures. It manifests across a varied clinical spectrum of expressions in children and young adults. It is crucial for children with OI to have a multidisciplinary follow-up, including orthopedics, pediatrics, and physical medicine and rehabilitation. Although exercise may have no effect on the disease itself, it might improve the autonomy, self-esteem, and fitness of these children.

Methods: Retrospective cohort analysis of children and young adults aged three or more years old followed-up in a Level III Pediatric Hospital between 1995 and 2020. Demographic and clinical data were obtained from the hospital records and from the caregivers via phone calls. To our knowledge, this is the first national case series published assessing exercise habits in children with this condition.

Results: Among the 21 patients studied, the median age was 14 years, with no gender predominance. Eighteen (86%) practiced regular physical activity, while the remaining three (14%), all of whom were type III OI, were totally dependent. Of the aforementioned 18 children, 12 (67%) considered practicing the same level of physical activity compared to their healthy peers, although most of them needed adaptations. The most reported extracurricular activity was swimming, in 50% of the cases. About 39% engaged in physical activity two times or less per week, and 89% practiced for one hour or less per session.

Discussion: Over the years, it has become clear that physical activity is an important part of OI management. While awareness of the importance of exercise already exists, proper planning, follow-up, and monitoring are essential.

## Introduction

Osteogenesis imperfecta (OI) is a monogenic hereditary connective tissue condition displaying diverse phenotypic manifestations, leading to bone fragility and osteopenia [1-3]. It is the most prevalent heritable bone fragility disorder in children, with an estimated incidence of one per 20.000 births, although milder forms are likely to be underdiagnosed [2,4,5]. There are no national registries for OI, but the experts believe that there are about two to three hundred patients in Portugal diagnosed and followed-up [6]. The OI clinical presentation is extremely variable and includes increased susceptibility to fractures, reduced bone mass, short stature, progressive skeletal deformities, bluish sclera, dentinogenesis imperfecta, ligament hyperlaxity, and hypoacusis [2,7].

Almost all individuals with OI have an autosomal dominant form that is caused by heterozygous mutations in one of the two genes encoding type I collagen, COL1A1 and COL1A2. However, in the past 10 years, defects in at least 18 other genes have been linked to the OI phenotype [4,5]. With the increased prevalence of genetic testing, mutations in these genes are more frequently identified in individuals prone to fractures but who do not have other typical clinical characteristics of OI. Mutations can be inherited from an affected parent or arise de novo [4].

The severity of bone fragility, and therefore the prognosis, varies widely, ranging from milder forms, that can manifest as premature osteoporosis, to severe ones with in utero manifestations [1,5]. Most patients have a moderate form, with greater bone fragility and fractures with minimal or no trauma, reaching adulthood [1]. It can be useful to categorize individuals with similar clinical characteristics into more narrowly defined OI types. Taking this into account, it was created a clinical classification, introduced by Sillence, which separates the severity spectrum of OI into four categories (OI types I to IV) [5,8]. OI type I represents the least severe phenotype, typically characterized by straight limbs and a body height within or slightly below the reference range [9]. OI type II is the utmost severity within the phenotypic spectrum, typically leading to intrauterine death or death shortly after birth, from respiratory failure. OI type III stands as the most severe manifestation of OI among individuals who survive the neonatal period. Those with OI type III typically experience restricted mobility and develop scoliosis. The disease severity of OI type IV is intermediate between OI types I and III. With proper care, the majority of individuals with OI type IV are able to walk, although more than half develop scoliosis [10]. Among the various OI types, OI type I is by far the most prevalent, comprising 70% of the entire cohort in a population‐based study [11].

While the most commonly used classification categorizes OI into four types, in 2015, the Nosology and Classification of Genetic Skeletal Disorders introduced a fifth OI type. This type is considerably rare and often exhibits skeletal features resembling those of OI type IV. However, OI type V is characterized by additional distinctive features, including the formation of hyperplastic callus (observed in approximately two-thirds of patients) and ossification of the interosseous membrane of the forearms (which eventually develops in almost all individuals with OI type V) [12].

Children with OI require multidisciplinary and specialized care, involving collaboration among orthopedics, pediatrics, and physical medicine and rehabilitation (PM&R) [13]. The goals of OI therapy include minimizing fracture occurrences, preventing and correcting bone deformities, minimizing chronic pain, and improving both mobility and functional independence [13]. The therapeutic strategy for individuals with OI is contingent upon factors such as age, disease severity, and functional status. Treatment encompasses optimizing calcium phosphate balance, rehabilitation therapies, orthotic adaptations, and, in certain instances, the consideration of bisphosphonates and surgical interventions [4,5].

In our hospital, all OI patients are integrated into a specific surveillance program that includes an initial assessment by different specialties such as pediatrics, orthopedics, physical medicine and rehabilitation (PM&R), otolaryngology, stomatology, cardiology, pneumology, neurology/neurosurgery, psychology, and palliative care (for more severe cases). The subsequent follow-up includes pediatric, PM&R, and orthopedic appointments every six months, stomatology annually, and otolaryngology, pneumology, and cardiology every two years. The monitoring of these patients also foresees the execution of some complementary diagnostic tests like calcium and vitamin D rationing, bone densitometry, audiometric test, electrography and echocardiography, and spirometry and electroencephalogram, with a periodicity that depends on the OI type. In most severe forms it must be excluded platybasia and craniovertebral impingement, with neurosurgery and MRI evaluation being obligatory in the early newborn/infant evaluation. The OI management is always individualized and may include a rehabilitation program in cooperation with the Portuguese OI Association, behavioral and lifestyle modifications (diet, exercise habits, and postural techniques), pharmacological interventions such as calcium and vitamin D supplementation, bisphosphonates in selected cases and orthopedic surgery.

Concerning the lifestyle nuances of OI patients, engaging in an appropriate amount of physical activity could potentially mitigate the adverse effects of aging on muscle function observed in these individuals [14]. Research indicates that while exercise may not directly impact the disease itself, it has the potential to enhance activities of daily living, self-esteem, and overall fitness in many children with OI [15]. The fear of fracturing may discourage individuals with OI from participating in physical activities, serving as a limiting factor to reaching their full physical potential [14]. For instance, children with uncomplicated OI type I might exhibit similar levels of physical activity as their healthy peers [5,14]. Nevertheless, there is a lack of studies on exercise habits in children with OI, and future research should focus on identifying perceived barriers to participation in physical exercise, both in the community and at school [15].

Therefore, with this study, we aim to describe the physical activity of children and young adults with OI, aged three or more years who have been followed-up at the OI outpatient clinic in our institution between 1995 and 2020. To our knowledge, this is the first national case series published assessing exercise habits in children with this condition. 

## Materials and methods

We conducted a retrospective cohort analysis of all children and young adults aged three or more years, followed by the OI appointment in a tertiary hospital in Lisbon, Portugal, between 1995 and 2020. The study was approved by the ethics committee. 

Out of the initial 38 patients traced back to 1995, 17 were excluded due to age constraints (being under three years old at the time of the study), incomplete medical records, or unresponsive to our follow-up phone call. The selection of a three-year-old threshold was strategic, aiming for a more objective evaluation of physical activity. This criterion considers the likelihood of a more regular and established exercise routine above this age, both within and outside of the school environment.

Our analysis delved into demographic factors (age, age at diagnosis, and gender) and clinical data (OI type, number of fractures until the present date, and therapeutic approach). With a focus on the physical activity performed, encompassing school-based and extracurricular exercises, the necessity for modified exercise, and details regarding frequency and intensity.

All data were collected through the examination of an established database, hospital records and nursing records. To assess the physical activity performance, we contacted all the caregivers via phone, with explicit verbal consent.

The obtained data were encoded and recorded in the Excel 2021® Software (Microsoft Corporation, New York, USA) and subsequently analyzed using the Statistical Software SPSS 24.0® (SPSS Inc., Chicago, IL). Data description relied on frequency distribution, measures of central tendency, and measures of dispersion. Non-parametric tests were employed for statistical analysis. Spearman and Pearson correlation tests were used to examine associations between two variables. A significance level of 5% was adopted for decision-making.

## Results

Of the selected 21 patients, 52% were male, with a median age of 14 years old, ranging from three to 28 years old. The median age of diagnosis was 24 months, with only two prenatal diagnoses. The genetic OI type was identified in 14 children: seven (50%) had type I, five (36%) had type III, and two (14%) had type IV. There was a statistically significant relationship between the type of OI and the median number of fractures (p = 0.021), with type III OI associated with a much higher number of fractures (10 to 100 fractures). 

About these patients’ management, the majority underwent at least one cycle of bisphosphonates therapy (n = 15; 71%) and were followed by a physical medicine and rehabilitation program (n = 17; 81%).

Almost all children (n = 18, 86%) practiced regular physical activity, 15 (71%) in school, 13 (62%) in the community, with three of them (14%) performing it exclusively as an extracurricular activity (water-adapted sports). The three patients (14%) who did not practice any kind of exercise were classified with OI type III and were totally dependent on caregivers for their daily basic activities (Tables 1, 2).

**Table 1:** Demographic and clinical characterization of the 21 OI patients y: years-old; mo: months; PA: physical activity; F: female; M: male; U: unknown; S: school; EC: extracurricular; NP: non-practicing; NA: not applicable; x/w: times per week; h: hour; min: minutes; OI: osteogenesis imperfecta. *At least one cycle of bisphosphonates during the course of the disease; †follow-up on a physical medicine and rehabilitation appointment; ‡ time per session for each type of physical activity; § perception of normal physical activity for their age, compared to their pairs.

	Demographic data			Clinical data				Physical activity data					
	Gender	Current age (y)	Age of diagnosis	OI type	Number of fractures	Bisphosphonates*	Rehabili-tation†	Where?	Sport?	Frequency	Intensity‡	Perception of normal PA?§	Adaptations?
1	F	9	Pre-natal	I	3	Yes	Yes	S + EC	Biking	S: 2x/w; EC: 1-2x/w	1 h each	Yes	Foam protections
2	M	12	11 mo	I	5	Yes	Yes	S + EC	Swimming	1-2x/w each	1 h each	Yes	Non-impact exercises
3	F	12	8 y	I	6	No	Yes	S	NA	2x/w	1 h 30 min	Yes	No
4	M	16	12 mo	I	8	Yes	Yes	S + EC	Swimming, gym	S: 2x/w; EC: 2x/w each	S: 1 h EC: 50 min + 1 h 30 min	Yes	Non-impact and gym-adapted exercises
5	M	7	2 y	I	13	Yes	Yes	S + EC	Hiking	S: 2x/w; EC: 1x/w	1 h each	Yes	No
6	M	15	11 y	I	6	Yes	No	S	NA	2x/w	1 h	Yes	Non-impact exercises
7	F	19	Birth	I	24	No	No	S + EC	Gym	S: 2x/w; EC: 3x/w	1 h each	Yes	Non-impact and gym-adapted exercises
8	M	16	2 y	III	10	Yes	Yes	NP	NA	NA	NA	NA	NA
9	M	13	Pre-natal	III	50-100	Yes	Yes	NP	NA	NA	NA	NA	NA
10	F	20	1 mo	III	12	No	No	S + EC	Boccia	S: 2x/w; EC: 2-3x/w	1 h each	No	On a wheelchair
11	M	27	Birth	III	30-50	Yes	Yes	NP	NA	NA	NA	NA	NA
12	F	8	Birth	III	>100	Yes	Yes	S + EC	Boccia, swimming	S: 2x/w; EC: 1x/w each	S: 1 h EC: 45 min + 30 min	No	On a wheelchair
13	F	17	2 y	IV	5	Yes	Yes	EC	Swimming, sailing	2-3x/w	1 h each	No	Adapted sailing
14	F	8	3 y	IV	8	Yes	Yes	S + EC	Swimming	2x/w each	S: 1 h; EC: 45 min	Yes	No
15	M	19	18 mo	U	26	Yes	Yes	EC	Swimming	2x/w	1 h	Yes	No
16	M	13	4 y	U	3	Yes	Yes	S + EC	Swimming	2x/w each	1 h each	Yes	Non-impact exercises
17	F	13	2 y	U	1	No	Yes	S + EC	Swimming	2x/w each	1 h each	Yes	Non-impact exercises
18	F	14	2 y	U	40	No	Yes	S	NA	2x/w	1 h	Yes	Non-impact exercises
19	M	20	2 y	U	30	Yes	Yes	EC	Swimming	<2x/w	<1 h	No	Adapted swimming
20	M	7	2 y	U	3	No	No	S	NA	2x/w	1 h	No	Non-impact exercises
21	F	18	18 mo	U	4	Yes	Yes	S	NA	2x/w	1 h	No	Non-impact exercises

**Table 2:** Physical activity according to the OI clinical type. OI: osteogenesis imperfecta.

	N	No physical activity	Physical activity	Swimming	Only at school	Boccia	Hiking	Cycling	Sailing	Gym
Type I	7	-	7	1	-	-	1	1	-	2
Type III	5	3	2	1	3	2	-	-	-	-
Type IV	2	-	2	2	-	-	-	-	1	
Unknown	7	-	7	4	5	-	-	-	-	-
Total	21	3	18	8	8	2	1	1	1	2

Of the 18 children that referred to regular physical activity, 12 (67%) considered practicing normal physical activity for their age and compared to their peers, but mostly with the necessary adaptations, such as the exclusion of impact activities or using foam protections to prevent fractures. The most performed extracurricular activity was swimming, in nine cases (50%), followed by boccia (two children, 11%, both with OI type III), gym (also two children, 11%, both with OI type I), hiking and cycling (both one patient with OI type I), and sailing (one patient with OI type IV). It should be noted that some children practiced more than one activity. Regarding the training frequency and intensity, seven children (39%) engaged in physical activity two times or less per week, and 16 (89%) practiced it for one hour or less per session (Tables 1, 2).

## Discussion

Until recently, parents were counseled to "shield" their children with OI by carrying them on pillows and avoiding recreational activities [16]. In fact, fear of fractures is a commonly cited reason for abstaining from sports and other physical activities, frequently advocated by parents and physicians in the pursuit of safety [3].

This protective attitude may inadvertently lead to an excessively sedentary lifestyle and physical deconditioning, impeding children's developmental progress and their attainment of independent functioning [3,16]. It is vital to note that bone growth relies not only on muscle pull but also on loading through weight-bearing activities like standing, walking, and lifting [16]. Engaging in physical activities is essential, as the positive influence of muscles on bone contributes to improved bone mineral density [17].

Over time, it has become evident that physical activity is a crucial component of OI management for both children and adults [16]. Nevertheless, children with mild to moderate forms of OI appear to face primary constraints related to diminished aerobic capacity and a reduced ability to generate muscle power [3]. The diminished aerobic capacity in individuals with OI is likely attributed to muscle atrophy and deconditioning, yet it remains uncertain whether this results from a hypoactive lifestyle or is a direct consequence of impaired muscle collagen synthesis [3]. However, an overprotective approach and insufficient muscle utilization may diminish the stimulus for bone formation, setting the stage for a potential "vicious cycle" involving "fracture-pain-fear of movement-immobility-deconditioning-reduced skeletal stability-fracture" [17]. Therefore, children and adults with OI can enhance their overall well-being through a consistent physical activity regimen, incorporating muscle strengthening, aerobic exercise, and recreational activities [16].

Engaging in a consistent regimen of physical exercise holds significant importance for nearly all our OI patients and caregivers, including those with the most severe forms of the condition.

We believe that the sample included in our study (n = 21), although small, is representative, taking into account the approximately 200 to 300 cases of OI diagnosed and being followed in Portugal among adults and children [6]. There was not a significant gender predominance, which is in accordance with its attributed genetic transmission: autosomal (dominant and rarely recessive) or with sporadic mutations [2]. Regarding the age at diagnosis, this usually varies with the subtype of the disease [2], with genetic and/or clinical characterization still pending in seven cases. Despite this, most type III OI patients were diagnosed in the neonatal period, in contrast to the milder-moderate forms, which had a later diagnosis.

Regardless of the minimal published evidence to support it, treatment with bisphosphonates at an earlier age appears to improve physical activity, particularly in severe forms of OI [18]. In our hospital, the selection for this treatment is based on an individualized and multidisciplinary decision, usually in patients over two years old, with moderate to severe OI, if the vertebral fracture or more than two long bones low-impact fractures in 12 months.

On OI, activity programs may include specific exercises recommended by rehabilitation professionals (physiatrists, physical therapists, occupational therapists, and recreation therapists) as well as sports and other recreational activities [16]. All of our patients were included in, at least, one of these activity programs, being the three patients who did not practice a regular sport or other recreational activity, and followed up on a physical medicine and rehabilitation program. 

Even so, it is recognized that many sports clubs, physical education classes, or extracurricular physical activities are not fully equipped to have children with this condition participating in their programs [3]. Nevertheless, most of our patients are considered to be capable of practicing normal physical activity for their age, compared to their peers, and reported feeling safe when performing it with the necessary adaptations, such as exclusion from impact exercises or using foam protections to prevent fractures. It is important that the rehabilitation team inform not only coaches and parents about the importance of sufficient and appropriate levels of exercise for these children but also other healthcare professionals, sports trainers, and teachers [3]. An environment that stimulates the motor development of the child and is also safe should be organized by parents, schools, and therapists [17]. This can increase physical activity and improve function and level of independence [17].

The extracurricular sports activity more frequently performed was swimming, which is the most recommended exercise for OI patients [7]. Considering the possibility of being adapted to all types of OI, even the most severe ones. It was empirically proven that children with OI can excel in the water, particularly if the activity is presented as an opportunity for recreation and independent exploration rather than a demand to exercise [16]. Water exercise can begin during infancy, and over time, the child can progress to independent activity in the water [16]. Walking in the water may even be possible for individuals who are unable to walk outside the pool, and water activities in childhood can be the foundation for a lifelong, enjoyable fitness activity [16].

As expected, patients with milder forms of OI were able to perform more complex, intensive, and apparently risky sports like gym, hiking, and cycling. In fact, for children and young people with OI types I and IV, it is recommended to engage in a physically active lifestyle, although contact sports and physical activities with sudden rotation moments of the joints are strongly discouraged [3,7,16]. Other authors take a more liberal view, stating that participation in activities involving high-impact or contact sports should be carefully assessed case by case, considering the specific activity and the individual [12]. Yet, the general recommendation for these children is supervised moderate aerobic training in combination with strengthening training without weights (or with less than 1 kg) [3,7]. Further studies are needed to address the safety and efficacy of exercise testing and training in children with other (more severe and/or wheelchair bound) types of OI [3]. 

Differences in muscular mass and strength are observed among children with OI, influenced by factors like OI type, age, and functional level. Therefore, an optimal training program should consider these variations and emphasize individual needs [7,17]. Currently, there are no universally accepted guidelines for the creation of training programs [3]. For chronic diseases like OI, it is recommended to use the so-called factors-frequency, intensity, time, and type-as a basic framework for developing an individual and/or disease-specific training program [3]. As variations between the different factors determine the efficacy of training, the key is to determine the most important contributing factors for each child [3]. In general, training frequency should be at least two times per week; intensity should be higher than 66% of peak heart rate, lasting for 20 to 60 minutes per session, in which large muscle groups must be used [3]. These training parameters are used in most formal sports activities. As we reported previously, in our cohort, seven children (39%) engaged in physical activity two times or less per week and 16 (89%) practiced it for one hour or less per session.

Our study presents some limitations associated with retrospective studies, namely the data collection of medical records, which may have led to the loss of information. The small size of the sample (n = 21) could also be a limitation. However, as explained previously, it should be considered a significant number of patients given the rarity of the disease. As information about OI type was not yet available in several cases, we could not draw more conclusions about the specificities of physical activity in each type of disease. We believe that future studies with OI patients should assess the more complete and safe exercises, the long-term effects of physical activity (regarding the number of fractures, level of independence, social participation, and improvement of life quality), and the impact of other therapeutic approaches (bisphosphonates, surgery) on these patients' physical performance. 

## Conclusions

Although there already seems to be awareness of the importance of exercise in OI management, it is imperative its planning, follow-up, and monitoring by those who follow OI patients, including clinicians, physical therapists, sports trainers, teachers and caregivers. This holistic approach ensures comprehensive care for OI patients.

While training programs for OI patients exist, there is a lack of universally accepted guidelines for personalized plans. Further studies are needed to establish clear guidelines for developing tailored training programs, improving quality of life, effective interventions, and safety for better outcomes. 
